# Aquatic Ecosystem Risk Assessment Generated by Accidental Silver Nanoparticle Spills in Groundwater

**DOI:** 10.3390/toxics11080671

**Published:** 2023-08-03

**Authors:** Rosember Ramirez, Vicenç Martí, R. M. Darbra

**Affiliations:** 1 Resource Recovery and Environmental Management (R2EM), Department of Chemical Engineering, Universitat Politècnica de Catalunya-Barcelona Tech, Diagonal 647, 08028 Barcelona, Catalonia, Spain; vicens.marti@upc.edu (V.M.); rm.darbra@upc.edu (R.M.D.); 2 Departamento de Ingeniería, Universidad Tecnológica del Chocó, Carrera 22 No.18B-10, Quibdó 270001, Colombia

**Keywords:** silver nanoparticles, aquatic ecosystems, Monte Carlo methods, fuzzy logic

## Abstract

This paper aims to create a new model for assessing the ecosystem risk in rivers and wetlands that are linked to accidental spills of silver nanoparticles (AgNPs) in soil/groundwater. Due to the uncertainty of the modeling inputs, a combination of two well-known risk assessment methodologies (Monte Carlo and fuzzy logic) were used. To test the new model, two hypothetical, accidental AgNP soil spill case studies were evaluated; both of which were located at the end of the Llobregat River basin within the metropolitan area of Barcelona (NE Spain). In both cases, the soil spill reached groundwater. In the first case, it was discharged into a river, and in the second case, it recharged a wetland. Concerning the results, in the first case study, a medium-risk assessment was achieved for most cases (83%), with just 10% of them falling below the future legal threshold concentration value. In the second case study, a high-risk assessment was obtained for most cases (84%), and none of the cases complied with the threshold value. A sensitivity analysis was conducted for the concentration and risk. The developed tool was proven capable of assessing risk in aquatic ecosystems when dealing with uncertain and variable data, which is an improvement compared to other risk assessment methodologies.

## 1. Introduction

Engineered nanoparticles (NPs) are used in the manufacture of many final consumer and intermediate goods. In particular, silver nanoparticles (AgNPs) have a wide range of new commercial and technological applications, which has led to increases in both their production and their release into the environment. AgNPs are used in various biomedical applications, such as wound healing, drug delivery, and cancer therapy [1,2]. AgNPs are also used in the textile industry to impart antimicrobial properties to fabrics [3]. In the same way, AgNPs are used in the electronics industry to produce conductive inks and coatings [4]. AgNPs are also used in water treatment to remove contaminants and to disinfect water [5].

These common applications inevitably lead to the continuous release of a fraction of these NPs into environmental compartments during their production, transportation, use, and processes of disposal, which have been extensively inventoried in many works [6,7,8,9,10]. Silver nanoparticles (AgNPs) are also some of the most-studied NPs due to their presence in European rivers, which is as a result of their continuous release [11]. AgNP concentrations below 1 ng/L are usually detected in rivers [12,13,14].

A very important topic is the accidental release of NPs into the environment; this subject is not considered in NP inventories, and risk experts have determined that it will become an important threat in the coming years [15,16]. Most of these accidental releases are spills; for this reason, soils are considered an important final destination for different types of NPs. NPs spilled onto soil can later reach groundwater, and, in specific cases, they may later impact other environmental compartments where aquatic ecosystems are present, such as lakes, rivers, and wetlands. As a consequence of these accidental spills, the acute exposure of aquatic ecosystems to NPs is expected.

The release of AgNPs into the environment, whether through continuous releases or accidental spills, can have adverse effects on organisms in aquatic ecosystems [9,16]. AgNPs have been found to be toxic to various aquatic organisms, including fish, crustaceans, and algae [17,18,19]. Even at low concentrations, AgNPs can have detrimental effects on these organisms, affecting their growth, reproduction, and overall health. AgNPs have the potential to bioaccumulate in organisms as silver species [20,21,22]. This can lead to the persistence of AgNPs in the environment for extended periods, resulting in long-term exposures of AgNPs and their potential accumulation in ecosystems.

For this reason, a new EU legislation proposal, the COM(2022) 540 final [23], has included Ag as a new priority substance with a maximum allowable concentration (MAC) of 22 ng/L in surface water. In order to study the impact of any accidental NP spills on soil, their potential arrival in groundwater, and their final impact on other aquatic ecosystems, a complete understanding of the fate and transport of NPs in subsurface environments is essential for developing strategies to assess the risk they present. Once NPs are in groundwater, they are subject to transport in this medium, which is usually described in terms of advection–dispersion and porous-media interaction mechanisms [24,25,26,27,28,29].

The transport of nanoparticles in porous media has received a significant amount of scientific interest in recent years due to its diverse applications, such as the remediation of groundwater with NPs or the use of porous media to filter water containing NPs. Several investigators have made advances in understanding the factors that influence the fate and transport of AgNPs in porous media [26,30,31,32,33,34,35,36] Most of these studies were performed using sand columns to represent the porous media, with concentrations ranging between 1 and 40 mg/L, and several studies have investigated the transport of AgNPs in natural soils [26,34,37]. Also, the mobilities of different types of coated AgNPs—such as NPs coated with citrate [33,38,39], PVP [26,35], or other substances [32,33]—were tested in these studies.

This kind of mobility information was used in an assessment of the risk of AgNPs linked to a contaminated aquifer by Tosco and Sethi [31], who simulated a human health risk assessment of AgNPs due to their possible arrival in groundwater from a landfill. Their results provide a framework for the application of a model that allows for risk assessment by using a simulation of the transport of NPs, one that considers the factors that affect the NPs’ interaction with the medium such as the size of the NPs, the type of porous medium, and the NPs’ coating.

Due to the complexity of the models needed to describe the transport of NPs and the inherent uncertainty associated with some properties used to determine the concentration and risk of NPs, a new approach can be used. This approach consists of integrating two models for risk assessment: Monte Carlo simulation and fuzzy logic. Several Monte Carlo simulations were recently applied to model the concentration of the NPs in wastewater [40,41] and environmental compartments [8], and a fuzzy logic approach developed by our group was used to assess the risk of AgNPs in aquatic ecosystems [41]. The combination of both models has been tested in the evaluation of the health risks associated with groundwater contamination [42,43], water contamination [44], and air contamination [45].

However, information on the accidents that lead to the arrival of NPs in surface water remains scarce. For this reason, the objective of this study is to evaluate, using a combination of the aforementioned methods, the risk of the presence of AgNPs in aquatic ecosystems from two accidental scenarios in which NPs spill onto soil and reach groundwater.

## 2. Materials and Methods

**Figure 1:**
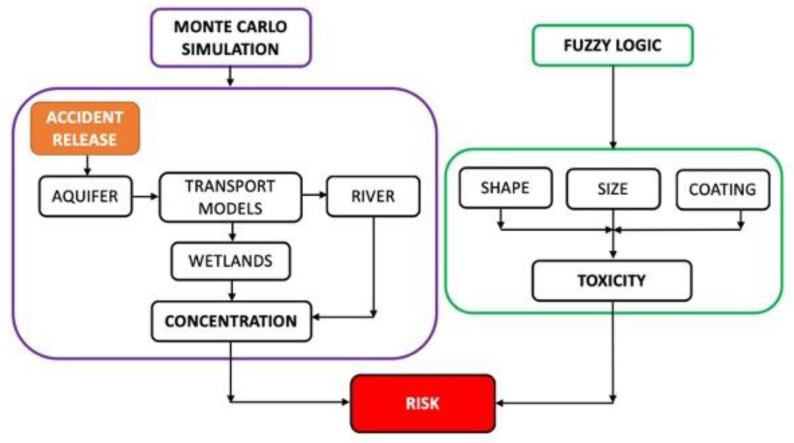
Schematic to illustrate the two models used: Monte Carlo simulation and fuzzy logic.

Figure 1 shows the approach of combining a Monte Carlo simulation and fuzzy logic to determine the maximum risk due to the presence of AgNPs in rivers and wetlands from their accidental release into groundwater.

The Monte Carlo approach was used to obtain a set of values for the maximum concentration of AgNPs in aquatic ecosystems, either rivers or wetlands, after their dilution in groundwater. On the other hand, the toxicity value was obtained from the application of the fuzzy model, which used a combination of the AgNPs’ properties (i.e., shape, size, and coating) to obtain this value. Finally, the risk was assessed by combining the concentration and toxicity through the use of the fuzzy model.

### 2.1. Modeling a Spill into a River or Wetland

In both case studies, the starting event is an accidental spill with a pulse (or instantaneous) discharge of AgNPs that forms a vertical linear source at x = 0 and y = 0. A rectangular domain of dimensions x, y, and z was considered in which x follows the direction of the groundwater velocity (considered only in this dimension), y is the horizontal direction perpendicular to the groundwater advance, and z is the vertical direction. Under these conditions, the model without a degradation of the contaminant could be applied for any z using the equation below [46]:

(1) $$C_{\left(x,y,t\right)}=\frac{m}{4\pi \cdot b\cdot n\cdot t\cdot \sqrt{D_{x}\cdot D_{y}}}\cdot \exp\left[-\frac{{\left(x-V_{\mathrm{fx}}\cdot t/F\right)}^{2}}{4D_{x}\cdot t/F}-\frac{y^{2}}{4D_{y}\cdot t/F}\right]$$

where:

m = Mass of pollutant injected per unit area (µg NP);

b = Length of the vertical source (m);

n = Porosity of the saturated medium;

t = Length of time since the spill (days);

V_fx_ = Linear velocity of the groundwater (m/day);

D_x_ = Longitudinal dispersion coefficient (m^2^/day);

D_y_ = Lateral dispersion coefficient (m^2^/day);

F = Retardation factor.

The Darcy expression was applied to the advection movement of the groundwater in the area that was in the direction of the gradient.

(2) $$V_{\mathrm{fx}}=\frac{q}{n}=\frac{k\cdot g}{n}$$

where:

k = Hydraulic conductivity of the saturated porous medium (m/day);

g = Average hydraulic gradient;

q = Average Darcy flow or Darcy velocity (m/day).

Since there was a vertical uniform distribution, there was no dispersion on the z axis, although there was a dispersion on the x and y axes, which is provided by the following equations:

(3) $$D_{x}={\;\alpha }_{x}\cdot V_{\mathrm{fx}}$$

(4) $$D_{y}={\;\alpha }_{y}\cdot V_{\mathrm{fx}}$$

where:

α_x_ = Longitudinal dispersivity (m);

α_y_ = Lateral dispersivity (m).

The longitudinal and lateral dispersivity values were calculated from the correlation of [47] as a function of α_x_, where x is in meters, as follows:

(5) $$\alpha_{x}=0.83\cdot {\left(\log x\right)}^{2.414}$$

(6) $$\alpha_{y}={\;\alpha }_{x}/10$$

In Equation (1), the concentration presents a Gaussian plane in two dimensions. The maximum concentration (C_max_) in the groundwater occurs on the axes y = 0 and z = 0, and at the time t = t_max_ when the value of x equals the distance L (the point of discharge into the river or the distance to the well) [46].

(7) $$L=\frac{V_{\mathrm{fx}}\cdot t_{\max}}{F}$$

A very detailed explanation of the retardation factor, F, in the case of the NPs can be found in Section 1 of the Supplementary Materials.

Using Equations (1), (3), (4) and (7), the following maximum value of C, C_max_ (ng/L), at a distance of L can be obtained as follows:

(8) $$C_{\max}=\frac{m}{4\pi \cdot b\cdot n\cdot t_{\max}\cdot \sqrt{D_{x}\cdot D_{y}}}=\frac{m}{4\pi \cdot b\cdot n\cdot F\cdot L\cdot \sqrt{\alpha_{x}\cdot \alpha_{y}}}$$

C_max_ was chosen because it is related to C_o_, i.e., the acute exposure of aquatic organisms to AgNPs.

In the first case study involving groundwater discharge, the Gaussian plane of interest is the one that cuts in at x = L at t = t_max_ since this is the point where the groundwater discharges into the river and reaches its maximum concentration. Beginning with Equation (1) and using Equation (8), the lateral distribution of the concentration can be calculated at L as follows:

(9) $$C_{\left(L,y\right)}=C_{\max}\cdot \exp\left[-\frac{y^{2}}{4D_{y}t_{\max}/F}\right]$$

This new equation is also a Gaussian plane that is independent of z. As we are in a time that corresponds to x = L (Equation (1)) in the previous equation, and by using Equations (4) and (7) from the text, we can obtain:

(10) $$C_{\left(L,y\right)}=C_{\max}\cdot \exp\left[-\frac{y^{2}}{4\alpha_{y}L}\right]$$

The width of the plume of AgNPs, W, in the y-direction for x = L can be assumed to be the distance covered by 95% of the Gaussian plane (twice the dispersion of y on each side). This value can be obtained as follows:

(11) $$W=4\sigma_{y}=4\sqrt{2\cdot \alpha_{y}\cdot L}$$

At this point, a section of groundwater A with a width W and a height b can be considered (as there is no dispersion in the z-direction). This allows for the calculation of the discharge area as follows:

(12) A=W·b

It is possible to calculate the average concentration in this section, C_D_, which will be independent of z and will only vary in the y-direction:

(13) $$C_{D}=\left(\frac{1}{W}\right)\cdot \int_{-W/2}^{W/2}C_{\left(L,y\right)}\cdot \mathrm{dy}\;$$

This integral resolves according to [48], and provides the following solution:

(14) $$C_{D}=\sqrt{\frac{\pi }{8}\cdot }\mathrm{erf}\left(\sqrt{2}\right)\cdot C_{\max}=0.5981C_{\max}$$

The discharge from the aquifer into the river occurs at the point x = L in the form of a flow, Q_D_ (m^3^/day).

(15) $$Q_{D}={\;q}_{d}\cdot A_{d}$$

where:

A_d_ = Connection area between the aquifer and the river (m^2^);

q_d_ = Darcy flow at the point of discharge (m/day).

The value of q_d_ does not have to match the average value of q, nor must A_d_ match with A. However, in the case of a one-sided discharge from the aquifer into the river at a steady state, a mass balance shows that the discharge at the point of discharge would be, on average, the same as the one that circulates through the aquifer (Q_A_), which can be replaced by the following equation obtained from Equations (2) and (11).

(16) $$Q_{D}=q\cdot W\cdot b\;=4k\cdot g\cdot b\cdot \sqrt{2\cdot \alpha_{y}\cdot L}$$

The concentration of NPs in the river, C_o_, can finally be obtained by performing a mass balance at the point of discharge:

(17) $$C_{0}=\frac{C_{R}^{\prime }\cdot Q_{R}^{\prime }+C_{D}\cdot Q_{D\;}}{Q_{R}}$$

where:

$C_{R}^{\prime }$ = Concentration of the AgNPs upstream from the source (ng/L or µg/m^3^);

$Q_{R}^{\prime }$ = River flow rate upstream from the source (m^3^/day);

$Q_{R}$ = River flow rate downstream from the source (m^3^/day).

Considering that there are no NPs in the river before the discharge point, and by using Equation (14), the following equation can be obtained:

(18) $$C_{0}=\frac{Q_{D}\cdot {\;C}_{D}}{Q_{R}}=\frac{0.5981{\;Q}_{D}\cdot C_{\mathrm{mx}}}{Q_{R}}$$

In the second case study, the value of C_max_ (Equation (8)) arrives at a well that feeds a wetland. The dilution of C_max_ in a wetland of a volume V allows for the estimation of the dilution factor (DF). The calculation of the DF is based on a mass balance in which the flow of the groundwater supplied to the wetland, q_w_, compensates for the water that is lost through evapotranspiration; therefore, the V of the wetland remains constant. The groundwater concentration, C_max_, is assumed to be maintained throughout the pumping time and is perfectly mixed with the volume of the wetland for a time t_m_ until an operator/manager realizes that the water is contaminated. Consequently, the maximum concentration in the wetland, considered to be the environmental concentration in the case of the wetland, C_o_, has a maximum value given by the following equation:

(19) $${\;C}_{o}=\frac{q_{w}\cdot C_{\max}\cdot t_{m}}{V}$$

The dilution factor (DF) can be expressed as follows:

(20) $$\mathrm{DF}=\frac{C_{0}}{C_{\max}}=\frac{q_{w}\cdot t_{m}}{\;V}$$

And thus, from Equations (19) and (20), the following may be obtained:

(21) $$C_{o}=C_{\max}\cdot \mathrm{DF}$$

### 2.2. Monte Carlo Model

A Monte Carlo model is a mathematical simulation technique used to estimate the probability of the outcomes of a variable and/or an uncertain event [49]. The Monte Carlo model has been successfully used in different areas related to NPs and environmental issues [50,51,52]. These contributions nicely demonstrate the use of the Monte Carlo model in studying and understanding the different sources of uncertainty and variability after a sensitivity analysis. Unlike a point estimation prediction model, a Monte Carlo model predicts a set of inputs, Z, and outputs Y results based on an estimated range of values [53]. This method creates a model of possible outcomes by taking advantage of a probability distribution of input values, such as a uniform or normal distribution, for any variable that has inherent uncertainty and variability [54]. The main steps implemented in the Monte Carlo methodology, and which are used in the present work, are as follows:

➢ The selection of the analytical models for the calculation of the dependent variables y (i.e., the maximum concentration of AgNPs in a river and/or wetland) as a function of input i and variables z_i_ (e.g., porosity, hydraulic conductivity, and river flow) (see Section 2.1) is as follows:

(22) $$y=f\left(z_{1},z_{2\;},z_{3},{\;z}_{4}\ldots .z_{i}\right)$$

➢ The determination of the probability distributions functions (PDFs) of the most relevant independent variables z_i_ (e.g., uniform, normal, and lognormal) is based on the knowledge of the variables and the case studies (see Section 2.4).
➢ The generation of N random values for each independent input variable z_i1_, z_i2_, z_i3_, …..z_iN_ is from each PDF. These results are organized in the vector Z_i_, which are formed by z_ij_ in which j is the random simulation order from 1 to N.

(23) $$Z_{i}=\left[z_{i1}{\;z}_{i2\;}z_{i3}{\;z}_{i4}\ldots .z_{\mathrm{iN}}\right]$$

In the present work, these input values correspond to porosity, hydraulic conductivity, the hydraulic gradient, the retardation factor, the river flow, and the dilution factor in the wetlands. Each parameter z_ij_ was calculated using a different random basis in order to simulate independent values:

➢ The application of the analytical models (see Section 2.1) for the simulation of the outputs (the maximum concentrations in the river and wetland) of the dependent variables for each random value j.

(24) $${\;y}_{j}=f\left(z_{1j},z_{2j\;},z_{3j},{\;z}_{4j}\ldots .z_{\mathrm{ij}}\right)$$

➢ The recalculation of the output results were conducted repeatedly using a different set of random numbers to produce an output vector Y (e.g., concentration).

(25) $$Y=\left[y_{1}\;y_{2}\;y_{3}{\;y}_{4}\ldots .y_{N}\right]$$

➢ The obtention of a probability distribution function of the dependent variables (i.e., the maximum concentration of AgNPs in the river and wetland) are given by Y. An analysis of the results (e.g., distribution shape and sensitivity analyses) (see Section 2.5) was also conducted.

### 2.3. Fuzzy Logic Model

Fuzzy logic is derived from traditional logic; however, it is based on the fact that while a statement does not have to be true or false, it will have to be verified to a certain degree [55]. Fuzzy systems describe uncertain phenomena and have been used in different applications [56,57,58], some of which are risk-related issues [59,60,61]. In fact, it can be said that this theory allows for the prediction of a risk situation where experimental data are difficult to collect, which is very useful.

**Figure 2:**
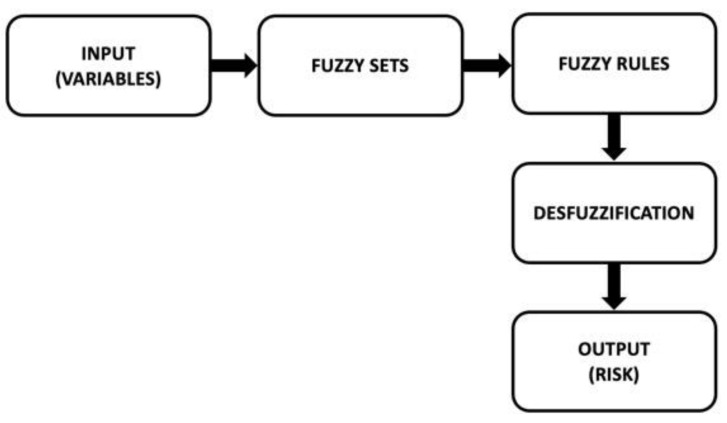
Schematic to illustrate the structure of the fuzzy logic model used.

MATLAB (v. R2020b, The Mathworks, Inc., Natick, MA, USA) and its Fuzzy Logic Toolbox (v. R2020b) were used to implement the fuzzy logic model. Figure 2 shows the main steps of implementing a fuzzy model.

As can be seen in Figure 2, the input variables are first established (e.g., pollutant concentration, toxicity, and risk). The fuzzy sets are then generated (e.g., high, medium, and low). The variables and fuzzy sets are then combined in rules (e.g., “if the concentration is low and the toxicity is high, then the risk is medium”). Finally, after a defuzzification process, an output value is obtained; in this study, the output is the risk value.

For the purpose of this study, a fuzzy model previously developed by the authors [41] was adapted and used to test the proposed case studies. From inputs such as the size, shape, and coating of the AgNPs, it is possible to determine their level of toxicity which, together with their concentration level, provides a risk assessment (see Figure 1). For more detail, see Table S2 in Section 3 of the Supplementary Materials, where the values of the variables used for the fuzzy model are shown.

### 2.4. Case Studies

The present work focuses on the low Llobregat River basin. This river is one of the most important fluvial axes in Catalonia. It is about 160 km long. Its source is in the pre-Pyrenees in Castellar de N’Hug, at an altitude of 1280 m, in the Sierra del Cadí, and it flows into the Mediterranean near Prat de Llobregat [62].

**Figure 3:**
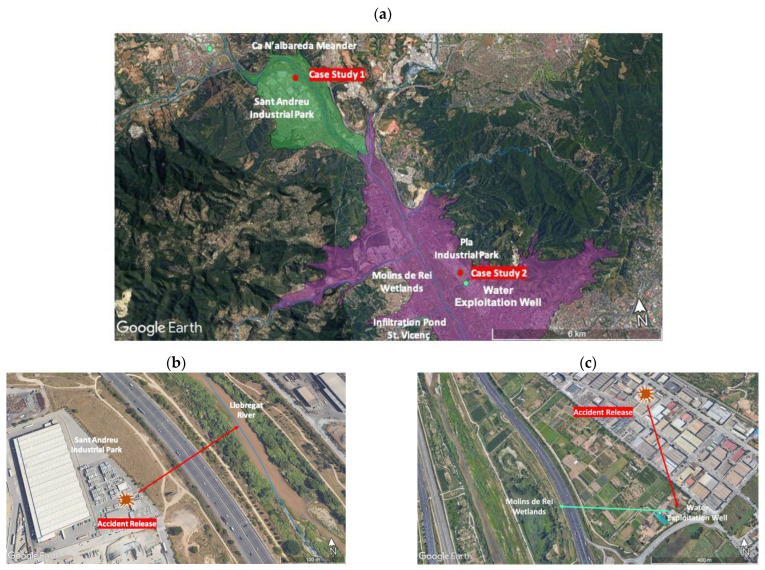
(**a**) Maps of the area for the two case studies. (**b**) Case Study 1: accidental release into groundwater, reaching the river. (**c**) Case Study 2: recharge of the wetlands with contaminated water from an accidental release.

The studied area ranges from the Sant Andreu Industrial Park to the Molins de Rei Wetlands. It is a vulnerable area with a high risk of infiltration and it discharges into the Llobregat River (Figure 3a) [63,64].

The figure also shows the groundwater water masses (green and violet) associated with the two sites. A simulation of two hypothetical, accidental releases of nanoparticles at two industrial sites was conducted: one located near a river and the other near a wetland (see both locations, labeled in red as Case Study 1 and Case Study 2, in Figure 3a). As a result of the accident, for each case, a total of 2 kg of solid-form silver nanoparticles entered the subsoil and reached the existing aquifer, filling the subsoil up to a depth of 3 m in both cases. The nanoparticles were subsequently transported over a distance of 200 m in the first case (the red arrow in Figure 3b) and 600 m in the second case (the red arrow in Figure 3c), finally reaching the river and the wetland, respectively.

The combination of the Monte Carlo simulation and fuzzy logic models was applied to quantify the maximum risk levels posed in the two case studies:

1- The presence of silver nanoparticles in the Llobregat River as a consequence of an accidental spill on the soil in the Sant Andreu Industrial Park; this spill reached the groundwater via its discharge and dilution in the Llobregat River (Figure 3b).
2- An accidental spill in another industrial park (Pla Industrial Park) reached the groundwater, and this was later used to recharge the Molins de Rei Wetlands (Figure 3c).

The released particles had a spherical shape and a size of 10 nm, and they were coated with citrate. A pulse (or instantaneous) mass input of AgNPs from a vertical line source, distributed within 3 m, was assumed. This discharge interacted with the porous media and suffered advection and dispersion.

Citrate was selected as the coating for the nanoparticles due to its broad applications and well-known effects on organisms. Citrate coatings are one of the most commonly used surface modifications for silver nanoparticles due to their stability and biocompatibility [65,66]. The citrate coating provides a negative charge on the surfaces of the nanoparticles, which can affect their behavior and transport in porous media [36]. Additionally, citrate-coated AgNPs have been widely studied, and their effects on various biological systems have been documented [39,67,68]. Investigating the effects of the citrate coating on the environmental fate and toxicity of AgNPs is crucial for assessing their potential risks.

In the first case study, the discharge reaches the river at a distance of 200 m from the source, and it is then diluted in the river to obtain a maximum concentration of AgNPs in the river (the mixing point).

In the second case study, the groundwater moves 600 m beneath an industrial zone (Pla Industrial Park), which is located to the NE of the Molins de Rei Wetlands, to a water exploitation well that is used to hypothetically feed the Molins de Rei Wetlands. The detailed models of the indirect discharge into surface water and wetlands via groundwater are explained in Section 2.1.

Table 1 shows the independent variables of the analytical equations that were modeled as constants or probability distribution functions (PDF). The procedure for the selection of values and probability distribution functions for each of the variables in the Monte Carlo approach, as applied to the two case studies, is explained below.

The first case study was located in a hydrogeological unit called the Cubeta de Sant Andreu de la Barca Basin (CSABB). This groundwater mass is marked in green in Figure 3a. Due to historical water extraction, this basin is one of the most studied basins in the area, but the quantitative hydrogeological data are old and scarce. The upper part of the CSABB (where the first case study is located) discharges into the Llobregat River beneath the previously mentioned industrial zone [69,70] (see Figure 3b).

According to well-established hydrogeological maps of this part of the Llobregat River basin [71], the natural materials of the studied aquifer comprise Quaternary materials formed by gravel and sand mixed with clay and/or silt materials. The average porosity reported in the aquifer in the CSABB is 0.15 [63], reaching 0.2 in other reports [64]. A probability distribution function commonly used for porosity in the literature is the normal distribution [72]. Therefore, an average value of 0.15 with ±2σ = 0.04 was chosen to cover the range from 0.11 to 0.19.

The reported average hydraulic gradient in the CSABB is 0.0001 [63], and the influences of water extraction and lateral recharge from nearby aquifers are important. This variability has been assumed to be uniform, and has been estimated to be ±50% of the average hydraulic gradient, ranging from 5*10^−4^ to 1.5*10^−3^.

The distribution of hydraulic conductivity in the saturated zone was estimated based on the described materials reported and on the data from two probes that were used to examine a pond in the Ca n’Albareda meander (see Figure 3a) [73].

The phreatic level was present at approximately 3 m below the ground, and the materials below were formed by gravel and sand. An estimation of the hydraulic conductivity from a measurement of granulometry revealed minimum values of around 80 m/day, as well as average values of 200 and 400 m/day for the most permeable materials of the probe [73,74]. The maximum values were similar to those provided in reports on the CSABB [63], which attributed hydraulic conductivity values of 100–300 m/d to this aquifer. As the most permeable materials mark the preferential path of groundwater, a range of 80 to 800 m/day with an average of approx. 250 m/day was considered. The probability distribution of hydraulic conductivity is usually modeled as a lognormal distribution according to [72]. Expressed in rounded logarithmic units, this corresponds to an average value of 5.5 and a standard deviation of 0.4, which covers the extreme values with 6σ.

In the second case study, the groundwater moves through an industrial zone (Polígon Industrial del Pla), which is located to the NE of the Molins de Rei Wetlands in the hydrogeological unit called the Cubeta de la Vall Baixa del Llobregat Basin (CVNLLB), (shown in purple in Figure 3a). In addition, it is connected downstream to the CSABB. In this second case, the groundwater flows in a parallel direction to the Llobregat River to a water exploitation well that is used to, hypothetically, feed the Molins de Rei Wetlands.

In this zone, Quaternary materials formed by gravel and sand mixed with clay are present. Following the same probability distribution type mentioned in the first case study, an average porosity value of 0.25 was reported in the CVNLLB [63], and a standard deviation of 0.02 was chosen with a final range between 0.21 and 0.29, based on 2·σ. For the hydraulic gradient, the same distribution and values as in Case Study 1 were chosen, as reported in [63].

Concerning the hydraulic conductivity, the data for the estimation were obtained from a sand aquifer located 7 to 7.5 m below the surface of an infiltration pond in St. Vicenç dels Horts [75,76], which is located only 2 km from the second case study (see Figure 3a). In these two references, an infiltration well and control piezometers were also used to measure the time of horizontal travel through the aquifer. From these travel times and the distances between the wells, groundwater velocities were estimated to range from 16.5 to 45.9 m/day. Through using the central values of porosity and the groundwater gradients measured in the assay (ca. 7–8*10^−3^), the values of hydraulic conductivity ranging from 480 to 1640 m/day were estimated in this aquifer. The reports of the CVNLLB [63] attributed hydraulic conductivity values of >100 m/day to this aquifer. Gathering this information, the chosen mean was around 480 m/day, with the extremes of ±3σ reaching 140 and 1640 m/day. Expressed in a natural log scale, the mean value was 6.1, and the standard deviation was 0.4. A detailed calculation of Q_R_ can be found in Section 2 of the Supplementary Materials.

The Molins de Rei Wetlands, which are artificial wetlands, extend over 6.8 Ha and have a minimum depth of 30 cm, indicating a volume of V = 20,000 m^3^ [77]. The use of impacted groundwater, which is pumped from nearby wells located in an industrial park, was considered. This water supply must balance evapotranspiration, which is about 3 mm/day or 30 m^3^/ha·day in this zone (Baix Llobregat) [78]. This requires a minimum of q_w_ = 204 m^3^/day or 8.5 m^3^/h from a depth of 30 cm.

In the accident considered herein, from the time t_m_ until somebody realizes the impact of the accident and stops the pumping, the pumping was uniform and moved from 1 h to 65 h (over an entire weekend). Therefore, through using Equation (20), the DF will be a uniform variable, ranging from 4.2*10^−4^ to 2.7·10^−2^.

The PDFs and constants were input into a MATLAB code (R2022a version, Mathworks^®^) using normalized random functions (uniform, normal, and lognormal) that were later scaled. In order to perform the Monte Carlo simulation, 10,000 values of the five random functions (n, F, g, k, Q_R_, or DF) in separate programs were obtained in a vector form Z_i_. In the case of F, a uniform PDF with a minimum degree of particle interaction with the porous medium of the groundwater (Fmax = 1.1) was chosen.

### 2.5. Sensitivity Analysis

The results of the Monte Carlo simulations were used to perform a one-dimensional Monte Carlo analysis (1D-MCA), for which output y (concentration, C_o_) was evaluated as a function of a single input z_i_. The scatter plots of these variables allowed for a first assessment of the sensitivity of the variables from Table 1 to be conducted. The relationship of y vs. z_i_ was quantitatively evaluated using the Spearman rank correlation r_s_ [79,80,81]_._ The value of r_s_ is a non-parametric type of correlation that is based in the comparison of the rank order of y and z_i_. The calculation of r_s_ was performed using the MATLAB command [rho, pval] = corr (Y’,Zi’,type = “Spearman”), where rho is r_s_. In the present study, ranking the values of r_s_ allowed for the determination of the variables that contribute the most to the concentration estimates.

For the evaluation of risk sensitivity, a simple approach using a sensitivity ratio (SR) was used [41]. A SR is a common metric of sensitivity analysis and, for quantitative parameters, measures the change in model output per unit change in an input variable.

In the present work, the sensitivity (SR_RC_) was assessed using small concentration intervals from which the ΔR/ΔC slopes were obtained, where R is the risk and C is the concentration. These slopes can be measured around a point (C_o_ and R_o_), and can be related to SR_RC_ using the following equation from reference [79]:

(26) $$SR_{RC}=\left(\frac{\Delta R}{\Delta C}\right)\cdot \frac{C_{0}\;}{R_{0}}$$

## 3. Results and Discussion

This section presents the results of the application of the combination of the Monte Carlo simulation and fuzzy model for each case study. For each case study, the results of the concentration and risk assessment are presented, in addition to a sensitivity analysis for both parameters.

### 3.1. Case Study 1

#### 3.1.1. Concentration

**Figure 4:**
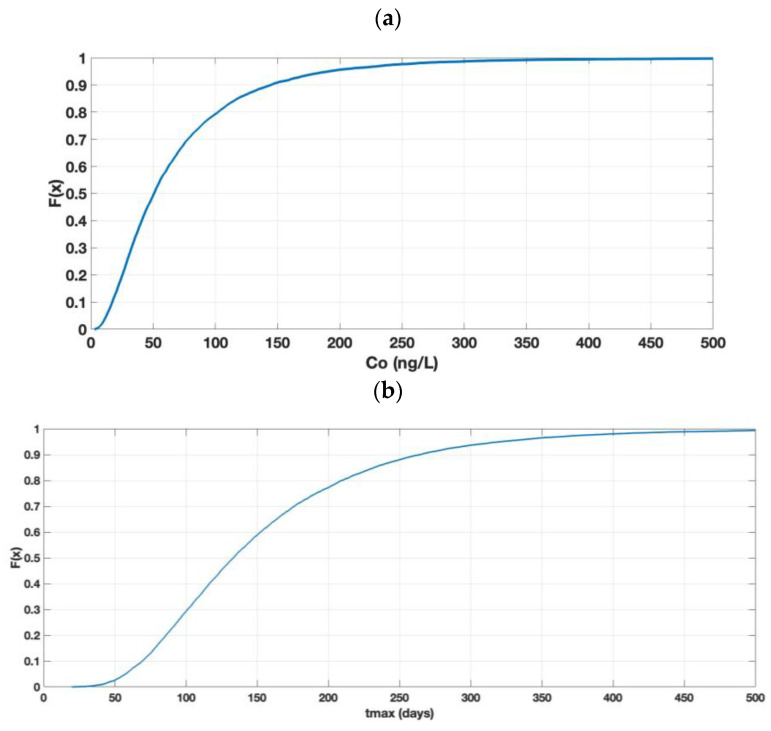
Results of the simulation of Co (rivers). (**a**) CDF of Co and (**b**) CDF of t_max_.

The results of the Monte Carlo simulation of C_o_ (river) for a spill of AgNPs, using the parameters of Table 1, are shown in Figure 4a as cumulative probability distribution curves (CDF) that are given by the function F(x), which moves in the range from 0 to 1. In addition, the Co values range from 0 to 500 ng/L, and these values originate from the simulation of different concentrations of AgNPs in the river. The C_o_ results show that, in 50% of the cases, the concentration present in the river was below 50 ng/L; in 95% of the cases, the concentration was below 200 ng/L.

Figure 4b shows t_max_, the groundwater transport time at which the concentration of the nanoparticles reaches its maximum value in the groundwater (C_max_) and in the river (C_o_). The figure shows that, in 50% of the cases, the AgNPs take less than 133 days to reach the river, with a minimum time of 19 days. In 90% of the cases, it took less than 266 days to reach the river.

As can be seen in Figure 4a, only approximately 10% of the simulation cases will comply with the new EU legislation COM(2022) 540 final [23] with values that are below 22 ng/L (MAC).

The simulated C_max_ values for this case study (not shown) indicate that approx. 50% of the cases were below 0.85 mg/L and 95% of the cases were below 1 mg/L. These C_max_ values (maximum concentration in groundwater) were lower than 1–40 mg/L in terms of AgNPs, i.e., the range of concentrations tested in column experiments [34,35,36,38], thus supporting the model’s hypothesis with respect to pseudo-linear models because the concentrations of the AgNPs in groundwater and those adsorbed in porous media (S_i_) were very low. Moreover, the blocking and ripening models converged with the linear model.

#### 3.1.2. Risk Assessment

**Figure 5:**
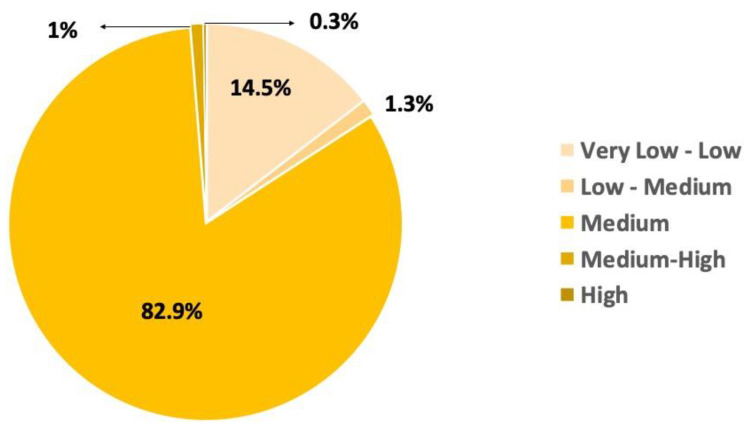
Distribution of the risk frequency for Case Study 1.

Figure 5 shows the results of the risk frequency simulation of the spill for citrate AgNPs. The values shown in the figure represent a percentage distribution of the cumulative distribution function (CDF) of the risk simulation, calculated from Figure S2a, which is presented in Section 4 of the Supplementary Materials. A relationship was presented between the different levels of risk in which the medium level of risk predominated.

#### 3.1.3. Sensitivity Analysis Applied to AgNP Concentration

**Figure 6:**
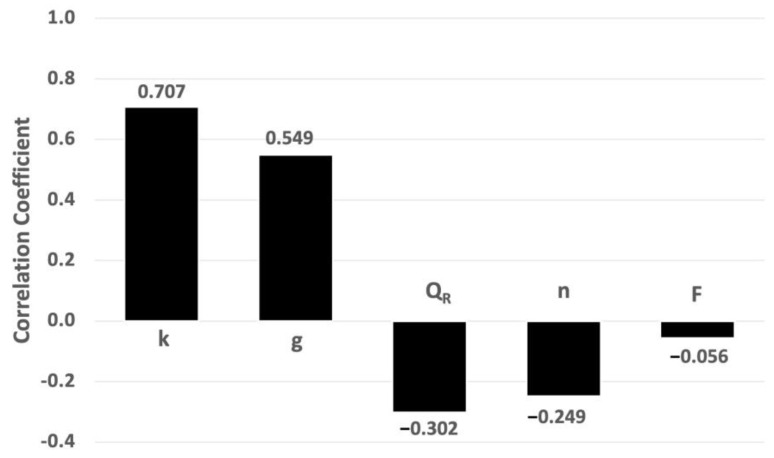
Sensitivity chart based on Spearman rank correlation coefficients (Case Study 1).

The degrees of sensitivity associated with the porosity (n), gradient (g), conductivity (k), delay factor (F), and the river flow rate (Q_R_) were studied. Figure 6 shows the sensitivity chart, which was determined using the Spearman rank correlation coefficients (r_s_) between Co and the five input variables. The higher the absolute value of r_s_, the higher the sensitivity. It can be observed that the conductivity (k) and gradient (g) had the greatest influences, followed by the river flow rate (Q_R_), porosity (n), and—finally—the delay factor (F).

These results reveal that the uncertainty linked to the discharge flow Q_D_ (specifically the Darcy flow, q) and river flow were paramount variables in the first case study. The parameters n and F, linked to C_max_ (or C_D_) according to Equation (8), had less influence.

Scatterplots from the 10,000 simulated values of Co as a function of k and g (see Section 5 of the Supplementary Materials) show a representation of the high correlation observed and the variation in these parameters.

A sensitivity analysis for the risk issue is discussed later in Section 3.2.4.

### 3.2. Case Study 2

#### 3.2.1. Concentration

**Figure 7:**
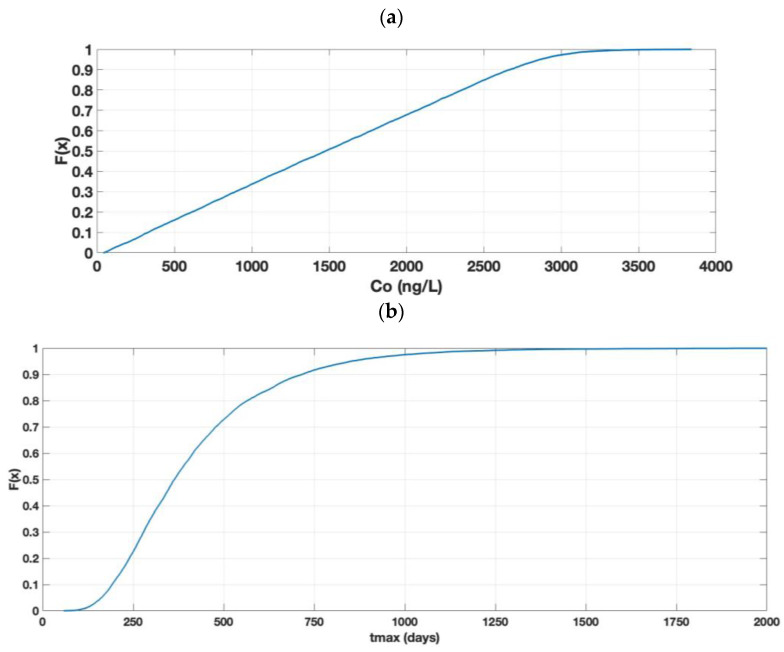
Results of the simulation of Co (wetlands). (**a**) CDF of Co and (**b**) CDF of t_max_.

The results of the simulation of Co (wetlands) for an AgNP spill, using the parameters of Table 1, are shown in Figure 7a as CD. Also, the Co values range from 0 to 4000 ng/L; these values originate from the simulation of the different concentrations of AgNPs in the wetlands. The Co results show that, in 50% of the cases, the concentration present in the wetlands was below 1500 ng/L; in 95% of the cases, it was below 3000 ng/L. In this case, the MAC value of 22 ng/L was surpassed in all cases because of the minimum value of 41 ng/L, as shown in Figure 7a.

Figure 7b shows t_max_, which is the groundwater transport time at which the concentration of the nanoparticles reaches its maximum value in the groundwater (C_max_) and in the wetland (C_o_). As shown in the figure, in 50% of the cases, it took less than 365 days (1 year) to pass through the aquifer and reach the well, with a minimum value of 58 days. The same figure shows that, in 90% of the cases, the time was less than 717 days.

The quasi-linear shape of Figure 7a is consistent with what is expressed in Equations (8) and (21), where it can be seen that C_o_ depends on the dilution factor (DF), retardation factor (F), and porosity (n). The first two functions are uniformly distributed, and the last one is normal. If the uniform functions were relevant, the expected representation would be linear. The simulated C_max_ values for this case study (not shown) were such that 50% of the cases were below 0.11 mg/L and 95% of the cases were below 0.13 mg/L. Again, the low C_max_ values support the pseudo-linear models, as in the first case study. The narrow variability of the p50 and p95 C_max_ values indicate a low degree of sensitivity in the groundwater parameters.

#### 3.2.2. Risk Assessment

**Figure 8:**
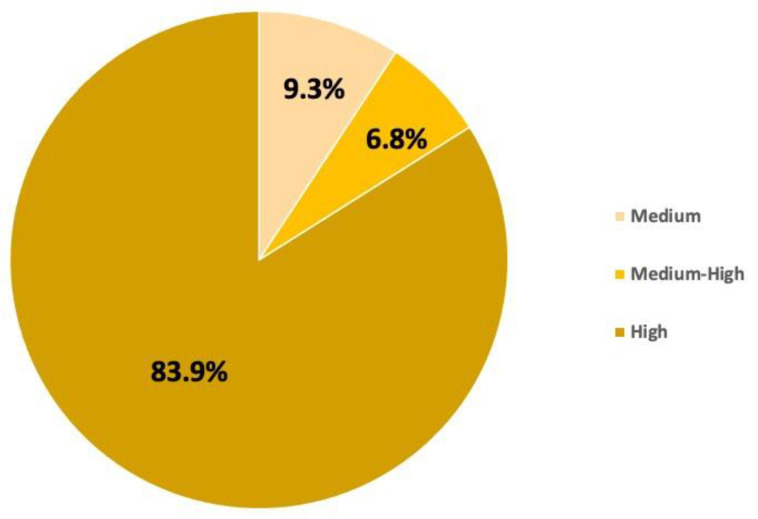
Distribution of risk frequency for Case Study 2.

Figure 8 shows the results of the risk frequency simulation. The values shown in the figure represent a percentage distribution, calculated from Figure S2b, of the cumulative distribution function (CDF) of the risk simulation, which is presented in Section 4 of the Supplementary Materials. In this case, only three levels of risk were presented, unlike the first case study for which a low-risk category (22 ng/L) was obtained. In most cases, the risk was high, as can be seen in the figure below.

#### 3.2.3. Sensitivity Analysis Applied to AgNP Concentration

**Figure 9:**
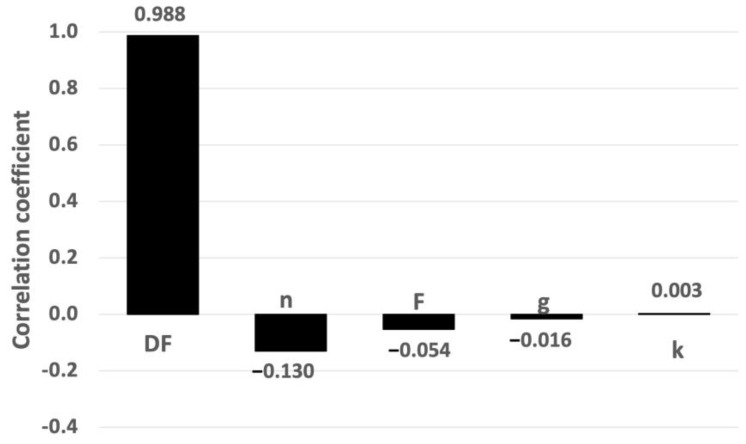
Sensitivity chart based on Spearman rank correlation coefficients (Case Study 2).

In the second case study, the sensitivity analysis focused on the same four first parameters as in Case Study 1 (the porosity (n), gradient (g), conductivity (k), and the delay factor (F)), in addition to the dilution factor (DF). Figure 9 shows the Spearman rank correlation coefficients between C_o_ and the input variables. It can be observed that the dilution factor (DF) had the greatest influence, followed by the minimal influences of porosity (n) and the delay factor (F).

**Figure 10:**
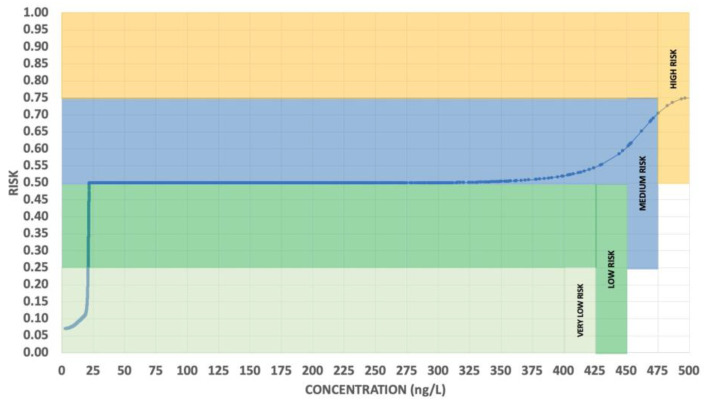
Fuzzy simulation of risk vs. Co.

These results show that the uncertainty linked to t_m_ (the time response) is a very important factor, and the parameters n and F—linked to C_max_—were much less influential. According to the theoretical expression of C_max_ (see Equation (8)), g and k had no influence; therefore, the very low results of these parameters, shown in Figure 10, validate the Spearman rank correlation coefficient methodology.

Scatterplots from the 10,000 simulated values of Co as a function of the DF and n (see Section 5 of the Supplementary Materials) provide a representation of the high correlation observed for DF and the very low correlation of n. In the case of the DF, a linear correlation was fitted (determination coefficient R^2^ = 0.9697), and this indicated a slope of 1.1*10^5^ ng/L, which is representative of the p50 of the C_max_ (0.11 mg/L) of the second case study (according to Equation (21)).

#### 3.2.4. Sensitivity Analysis Applied to Risk Assessment

Herein, the risk is a function of toxicity and concentration. Toxicity depends on the size, coating, and shape of the AgNPs (see Figure 1); however, only the size was assessed in terms of sensitivity since the values for coating (citrate) and shape (spherical) were considered certain. A 50% size variation (5–15 nm) was applied. However, the results from the fuzzy functions show that the sensitivity of the toxicity in relation to the AgNPs’ size in the studied range (5–15 nm) was zero (insensitive). This result agrees with the sensitivity analysis for the same type of nanoparticle that was conducted by the authors of [41], who focused their attention for the sensitivity analysis on the concentration of AgNPs.

Figure 10 shows the relationship between risk and C_o_ due to the variation in the concentrations of AgNPs for the citrate spheres. The figure begins from very low to low levels of risk below 22 ng/L, and then shows a medium level of risk for concentrations between 22 and 375 ng/L. From here, the risk increases to a maximum level for the concentrations of 500 ng/L (75–100% high risk), and, from here, there are no variations due to a limitation imposed by the fuzzy rules on toxicity.

The slopes in Figure 10 for each C_o_ and R_o_ were used to assess, using Equation (26), the sensitivity for risk as a function of the studied input variables. In this figure, concentrations from 22 to 300 ng/L, and those higher than 490 ng/L, presented zero values for the slope and SR. The rest of the SR_CR_ values were calculated and are summarized in Table 2. In the first case study, the most sensitive cases were concentrated in a very narrow interval (19–22 ng/L), with an extreme value of SR_CR_ = 21.3 at 21.25 ng/L and a sudden SR_CR_ = 0 at 22 ng/L. A second maximum of SR was concentrated in the range of 430–475 ng/L, reaching a maximum of SR = 3.1 at 454 ng/L. After 475 ng/L, the SR value decreased to zero at 490 ng/L. In Case Study 2, the risk begins from 41 ng/L, eliminating the first sensitive point described in Case Study 1. This shows an identical behavior to Case Study 1 for the risk sensitivity, with a zero slope from 41 to 300 ng/L and for concentrations that were higher than 490 ng/L. These results show that only the parameters used to determine sensitivity in groundwater, rivers, and wetlands (explained in Section 3.1.3 and Section 3.2.3) have a large influence on the risk for the highest value of sensitivity (SR).

## 4. Conclusions

This work presents a new methodology for evaluating the accidental risk posed by AgNPs for aquatic ecosystems by using two well-known and established models: Monte Carlo and fuzzy logic. To test the tool, two different case studies were used, both of which were based on accidents with the same amount of AgNPs spilled (2 kg) and with an equal distribution in soil (3 m); these AgNPs also reached groundwater and later a river or a wetland.

The available data for the aquifers, specific sites, rivers, and wetlands were evaluated and converted into Monte Carlo input variables to finally determine the probability distribution for the maximum concentration of AgNPs (acute effect) that could arrive in the rivers or wetlands as a consequence of the spill. This distribution of the concentration was introduced in a fuzzy model to finally obtain a risk assessment evaluation for the introduction of AgNPs into the aquatic ecosystems.

The outcomes of the study reveal that, for Case Study 1, the risk was mainly at a medium level, whereas for Case Study 2, in the majority of cases, the risk was high. Concerning the concentrations achieved due to the spill, in the first case study, 10% of the values were below the future legal limit, whereas in the second case study, none of them complied with legislation. Therefore, the impact of the spill of AgNPs that reached the river had less severe consequences than the use of polluted groundwater to recharge the wetlands.

In this study, risk was measured as a function of concentration and toxicity. Therefore, the sensitivities of these parameters were studied. For the case of concentration, Spearman rank correlation coefficients between Co and the different input variables were assessed. For Case Study 1, the conductivity, porosity, and river flow were the parameters with the greatest influence, whereas for Case Study 2, the dilution factor had the greatest influence. The sensitivity related to toxicity mainly depends on the size of the particle, since the coating and shape do not vary. However, the sensitivity study showed that the toxicity was insensitive to a size in the range of 5–15 nm; therefore, all the variation in the risk value came from the concentration. For the values between 22 ng/L and 300 ng/L, and which were above 490 ng/L, the risk did not vary (i.e., it was insensitive to concentration).

The methodology developed was proved to be capable of assessing risk by using uncertain data, and this was because it is based on a hybrid method between the Monte Carlo and fuzzy logic theories, which—when combined—can overcome the variability in the data inputs. In addition, the new method can be adapted to different scenarios and types of AgNPs, as well as other nanoparticles. This methodology is easy to modify, and the connection between the two theories is automatic. In the future, the risks of other engineered nanoparticles (e.g., TiO_2_, CeO_2_, and ZnO) or new emergent pollutants in aquatic ecosystems, such as microplastics, can be assessed using this method.

## Figures and Tables

**Table 1:** Variables and distribution parameters used for the Monte Carlo modeling in the two case studies.

	Variables z_i_ (Units)	PDF Type	Distribution Parameters	
			Accident from Case Study 1	Accident from Case Study 2
Source	m (µg)	Constant	2*10^9^	
Aquifer	b (m)	Constant	3	
	L (m)	Constant	200	600
	n (-)	Normal	Mean = 0.15 St. dev. = 0.02	Mean = 0.25 St. dev. = 0.02
	g (-)	Uniform	Min = 0.0005 Max = 0.0015	
	k (m/day)	LogNormal	Mean = 5.5 St dev. = 0.4	Mean = 6.1 St. dev. = 0.4
Interaction NPs—Aquifer	F (-)	Uniform	Min = 1 Max = 1.1	
River	Q_R_ (m^3^/s)	LogNormal	Mean = 1.887 St. dev. = 0.248	-
Wetland	DF (-)	Uniform	-	Min = 4.2*10^−4^ Max =2.7*10^−2^

Variables: m = the mass of the released AgNPs; b = infiltration depth; L = distance from the spill to the river or well; n = porosity; F = delay factor; g = gradient; k = hydraulic conductivity; St. dev. = standard deviation; Q_R_ = river flow rate upstream from the source; and DF = dilution factor in wetland (detailed in the text).

**Table 2:** Sensitivity ratio analysis.

		SR_XY_	
X-Y	C_o_ (ng/L)	Case Study 1	Case Study 2
Size *-T, Size *-R	-	0	0
C_o_-R	3–8	0–0.2	-
	8–12	0.2–0.5	-
	12–19	0.5–2	-
	19.0–21.9	2–21.3	-
	21.90–21.99	0.2–2	-
	22–300	0 **	
	300–370	0–0.2	
	370–400	0.2–0.5	
	400–430	0.5–2	
	430–475	2–3.1	
	475–490	0.2–2	
	>490	0	

* Size 5–15 nm. ** From 41 ng/L in Case Study 2.

## Data Availability

The data presented in this study are available upon request from the corresponding authors.

## Supplementary Materials

The following supporting information can be downloaded at: https://www.mdpi.com/article/10.3390/toxics11080671/s1, Figure S1: The fitting data for the calculation of the Llobregat River flow; Figure S2: CDF results of the risk simulation. (a) Case Study 1 and (b) Case Study 2; Figure S3: Scatterplots for Co in Case Study 1. (a) Hydraulic conductivity (k) 1 and (b) gradient (g); Figure S4: Scatterplots for Co in Case Study 2. (a) Dilution factor (DF) and (b) porosity (n). Table S1: The particles’ mechanisms of interaction with the porous medium; Table S2: The fuzzy sets, ranges, and types of membership functions (MFs) of the model variables.
