# Supplementary material for: Aquatic Ecosystem Risk Assessment Generated by Accidental Silver Nanoparticle Spills in Groundwater
Source: Toxics. 2023 Aug 3;11(8):671. doi: 10.3390/toxics11080671 (PMC10459696; doi:10.3390/toxics11080671)
Supplement: Supplementary file 1 [file toxics-11-00671-s001.zip › toxics-2457262-supplementary.pdf]

Supplementary materials

Aquatic Ecosystem Risk Assessment of Silver Nanoparticles accidental spills in groundwater

Rosember Ramirez*[1,2], Vicenç Martí[1] and R. M. Darbra[1]

[1]Resource Recovery and Environmental Management (R2EM). Department of Chemical Engineering, Universitat Politècnica de Catalunya. Barcelona Tech. Diagonal 647, 08028 Barcelona, Catalonia, Spain.; vicens.marti@upc.edu (V.M.); rm.darbra@upc.edu (R.M.D.)
[2]Departamento de Ingeniería, Universidad Tecnológica del Chocó, Quibdó, Carrera 22 No.18B-10, Colombia
*Correspondence: rosember.ramirez@upc.edu (R.R.); Tel: +34 934010811

1. Interaction of AgNPs with porous media and delay

Groundwater nanoparticles are dispersed, transported, and interact with the porous groundwater medium. Transport in this medium is usually described by a modified advection-dispersion equation that takes into account mass exchanges between the liquid and solid phases due to physical and physicochemical interactions [1]:

$$n \cdot \frac{\partial c}{\partial t} + \sum \rho_b \cdot \frac{\partial s_i}{\partial t} + q \cdot \frac{\partial c}{\partial x} - n \cdot D \cdot \frac{\partial^2 c}{\partial^2 x} = 0 \qquad \text{(S1)}$$

$$\sum \rho_b \cdot \frac{\partial s_i}{\partial t} = \sum n \cdot k_{att,i} \cdot f_{att,i} \cdot c - \rho_b \cdot k_{det,i} \cdot S_i \qquad \text{(S2)}$$

Where c is the concentration of nanoparticles in the liquid phase, S_i is the concentration of nanoparticles in the solid phase, $f_{att,i}$ is a term that depends on the interaction mechanism (see table S1), ρ_b is the apparent density of the porous medium, and D is the hydrodynamic dispersion coefficient, k_{att} and k_{det} are the adsorption and desorption rate coefficients of nanoparticles.

Table S1. Interaction mechanisms of particles with the porous medium.

Models	Description	Equations
**Clean bed filtration**	This mechanism considers first-order irreversible kinetics, while the desorption coefficient is considered negligible: k_{det} = 0. It mainly describes the attachment to the surface of the porous medium.	$f_{att} = 1$
**Lineal**	The linear colloid deposition mechanism is governed by the same principles as “clean bed filtration” but in this phenomenon, the desorption rate coefficient is not assumed to be negligible: $k_{det} > 0$	$f_{att} = 1$
**Blocking**	Where S_{max} is the maximum concentration of particles that can be retained in the solid phase under given chemical conditions [-], and is related to the surface blockage operated by already deposited particles.	$f_{att} = 1 - \frac{S}{S_{max}}$
**Ripening**	Where A_{rip} [-] y β_{rip} [-]are the maturation coefficients, where $A_{rip} > 0$ y $\beta_{rip} > 0$, so the deposition rate increases with increasing concentration of attached particles [2].	$f_{att} = 1 + A_{rip} \cdot S^{\beta_{rip}}$
**Straining**	where L is the path length of the particles in the porous medium and β_{srt} [-] is a fitting parameter that controls the shape of the spatial distribution of the particles. Experimental evidence has shown that β_{srt} can be taken equal to 0.432 with good results [2].	$f_{att} = \left(1 + \frac{L}{d_{50}}\right)^{-\beta_{srt}}$

The desorption term (k_{det}) is linearly proportional to the concentration of adhering particles. On the contrary, the function f_{att} in the adsorption term (k_{att}) is a generic function that depends on adsorption mechanisms such as clean bed, blocking, ripening, linear etc. The phenomenon of the delay in the mobility of the NPs is produced by their interaction with the porous medium. The type of mechanism depends, among other factors, on the coating of the particles, their size and the type of porous medium. The differential equations of main mechanisms are presented in the table S1. To solve these equations, finite element methods can be applied [3] or analytical equations are used in the case of the linear model, considering equilibrium. Thus, the retardation factor, F, in the case of nanoparticles that respond to the linear model can be described as [4]:

$$F = 1 + \frac{K_{att}}{K_{det}} \qquad \text{(S3)}$$

In this article, a pseudo-linear model has been chosen due to the short contact time of NPs with porous media and low concentration of AgNPs in groundwater (C_{max}), that allows the linearization of the other models. As it could be seen in Table S1, if contact time is short, the S values (adsorbed particles in the porous media) in blocking and ripening models are low and f_{att} is close to 1.

Straining model could be rejected, as the particles in the case study have a very low size (10 nm) compared with the pores of the porous media (sand and gravel). Clean Bed filtration model (irreversible attachment) could be also neglected due to short contact time. The advantage of this approach is that it allows to find ranges of these values for certain pairs of NPs-porous medium from several references and that it allows to apply the classical delay approach, already used by other authors [4].

2. Justification of Llobregat river flow input parameters for the Monte Carlo model

The modeling of the river flow distribution at the point discharge in the first case study has been derived from open data of Catalan Water Agency [5] about daily flow river in the stations of Martorell and Castellbisbal (see figure 3 a)) from 01/09/2020 to 01/09/2022, that is a period without extreme punctual high flow and, thus, representative of the accident. The flow data were sorted from lower to higher and a cumulative lognormal distribution was fitted [6] using nonlinear regression. To apply nonlinear regression, the Solver function in Microsoft Excel™ software with the GRG nonlinear solving method was used to minimize the SS_{err} as the objective function.

$$SS_{err} = \sum_{i=1}^{n} \left[Q_{data} - Q_{fit}\right]^2 \qquad (4)$$

where Q_{data} are the flows from available open data [5] and Q_{fit} is the fitting values given by the solver and n is the number of data. The graphical result of the fitting is shown in Figure S1. As it can be seen in Figure S1, a correct fitting was obtained and then is possible to represent the river flow as a Log Normal probability distribution function instead a temporal flow variation. As it could be seen, the distribution parameters expressed as natural logarithms are a mean of 1.887 and a standard deviation of 0.248.

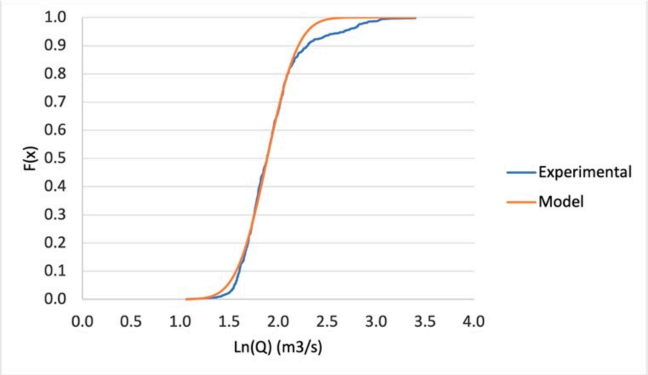

Figure S1. Fitting data for the calculation of the Llobregat river flow

These values representative of two years could be compared to lognormal distribution parameters used for Llobregat river in literature ([6]). In this reference, representative values of a period from 2005 to 2015, showed higher both mean and standard deviation values, that could be attributed to a wetter year and a higher dispersion due to 10 years period instead of the 2 years (this study).

3. Fuzzy Model

Table S2 shows the variables used in the fuzzy model presented in Figure 1 with their respective values. The ranges are presented for the different fuzzy sets as well as the functions used: Z shape for low values; Pi shape for medium values; and S shape for high values. This table is an adaptation of the fuzzy logic model used by [7]. In addition, with respect to the previous model, new categories for toxicity and concentration (very low in both cases) have been incorporated. In the case of concentration, the new category corresponds to the new EU legislation COM (2022) 540 final [8] which establishes a Maximum Allowable Concentration (MAC) of 22 ng Ag/L. For the present paper, the toxicity value is calculated for a single type of nanoparticle (i.e., AgNPs spheres, citrate coated and with a size of 10 nm). With this particle a value of 0.65 of toxicity is achieved using the adapted model. This value has been used for the two case studies and comes from the combination of the size, shape and coating of the particle. For more information for each one of latter variables, please check [7].

Table S2. Fuzzy sets, ranges, and types of membership function (MF) of the model variables.

Variables	Fuzzy Set	Ranges	MF types
**Toxicity***	Very Low	0 – 0.35	Z Shape
	Low	0.15 – 0.65	Pi Shape
	Medium	0.4 – 0.9	Pi Shape
	High	0.65 – 1	S Shape
**Concentration**	Very Low	0 – 22 ng L^{-1}	Z Shape
	Low	18 – 250 ng L^{-1}	Pi Shape
	Medium	150 – 500 ng L^{-1}	Pi Shape
	High	300 – 1000** ng L^{-1}	S Shape
**Risk***	Very Low	0 – 0.25	Z Shape
	Low	0 – 0.5	Pi Shape
	Medium	0.25 – 0.75	Pi Shape
	High	0.5 – 1	Pi Shape

Very High	0.75 – 1	Z Shape

* These variables are qualitative (without specific units). **For values higher than 1000 ng L^{-1} the function values are 1.

4. Risk assessment

Figure S2 shows the Cumulative Distribution Function (CDF) results of risk simulation with MATLAB for the two case studies. In *S2 (a) case study 1* the CDF results of risk simulation show that 15.85% of the cases represent a risk below 0.5 and almost the rest (82.87%) a risk equal to 0.5. In the upper part of the graphic, there is a very low accumulated percentage (1.28%) that has a risk from 0.5 to 0.75. Then in *S2 (b) case study 2,* the CDF results of risk simulation show that 16.04% of the cases represent a risk below 0.75 (medium or medium-high) and almost the rest (83.96%) a risk equal to 0.75 (high).

S2 (a) S2 (b)

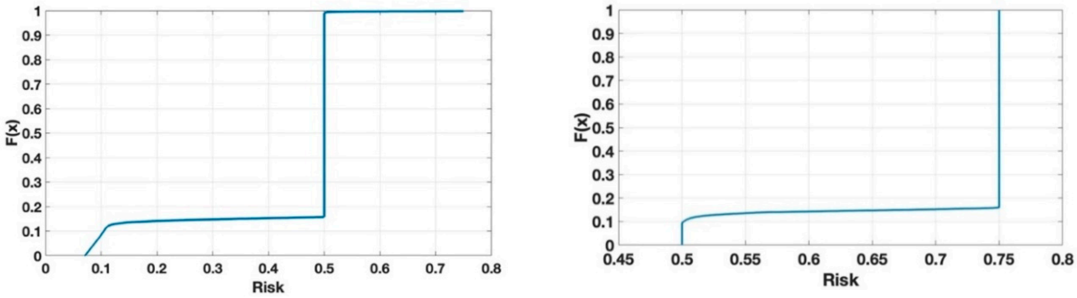

Figure S2. CDF results of risk simulation. S2 (a) case study 1 and S2 (b) case study 2.

5. Sensitivity analysis applied to AgNPs concentration.

5.1. Case study 1

Figure S3 shows the scatter plots for two variables are presented for the case de study 1. These are the two variables that showed the greatest influence for the concentration C_o.

S3 (a)

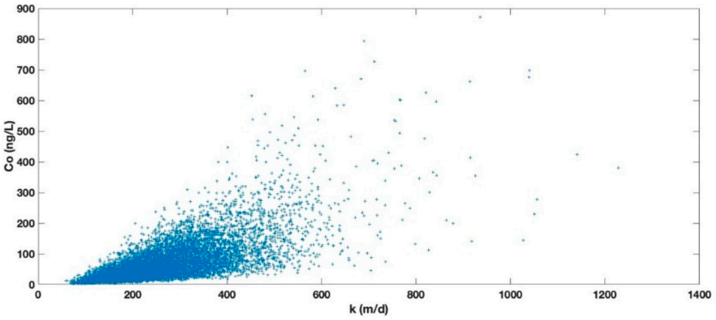

S3 (b)

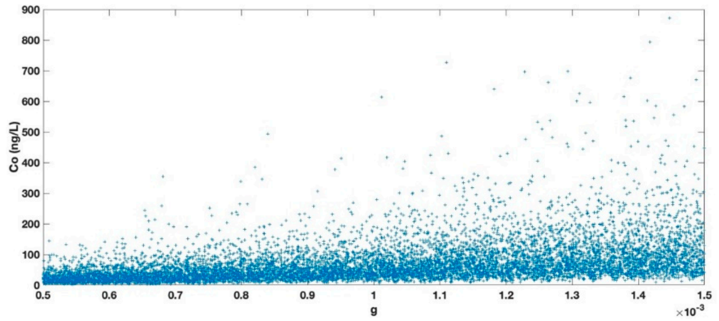

Figure S3. Scatterplots case study 1. S3 (a) Hydraulic conductivity (k) 1 and S3 (b) Gradient (g).

5.2. Case study 2

Figure S4 shows the scatter plots for two variables are presented for the case de study 2. These are the variables that have the greatest influence for the concentration, C_o.

S4 (a)

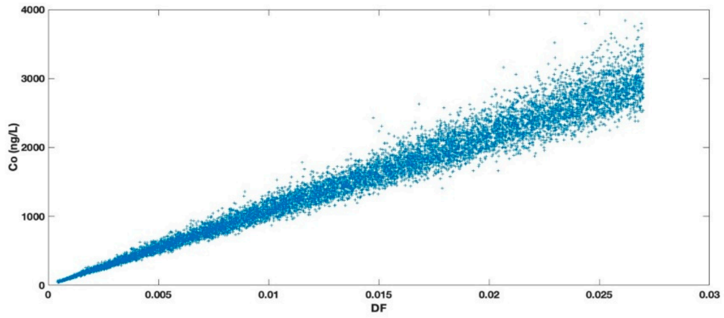

S4 (b)

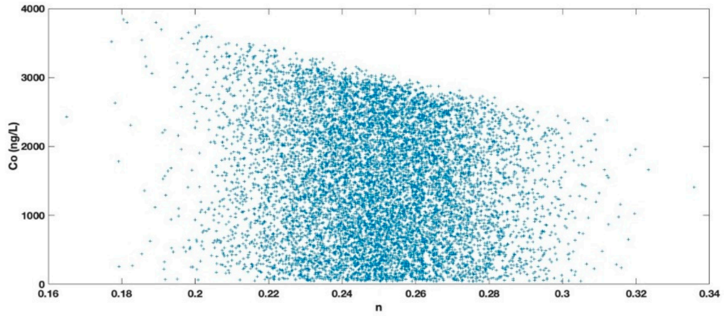

Figure S4. Scatterplots case study 2. S4 (a) Dilution Factor (DF) 1 and S4 (b) porosity (n).

References

1. Tosco, T.; Sethi, R. MNM1D: A numerical code for colloid transport in porous media: Implementation and validation. *Am. J. Environ. Sci.* **2009**, *5*, 516–524, doi:10.3844/ajessp.2009.516.524.
2. Bradford, S.A.; Simunek, J.; Bettahar, M.; Van Genuchten, M.T.; Yates, S.R. Modeling colloid attachment, straining, and exclusion in saturated porous media. *Environ. Sci. Technol.* **2003**, *37*, 2242–2250, doi:10.1021/es025899u.
3. Bianco, C.; Tosco, T.; Sethi, R. A 3-dimensional micro- and nanoparticle transport and filtration model

(MNM3D) applied to the migration of carbon-based nanomaterials in porous media. *J. Contam. Hydrol.* **2016**, *193*, 10–20, doi:10.1016/j.jconhyd.2016.08.006.
4. Tosco, T.; Sethi, R. Human health risk assessment for nanoparticle-contaminated aquifer systems. *Environ. Pollut.* **2018**, *239*, 242–252, doi:10.1016/j.envpol.2018.03.041.
5. Agencia Catalana de l’Aigua Consulta de les dades de control de la qualitat i la quantitat de l’aigua al medi Available online: http://aca-web.gencat.cat/sdim21/seleccioXarxes.do;jsessionid=4B6442745A6C3695855A89488EBD1B3F (accessed on Oct 12, 2022).
6. Banjac, Z.; Ginebreda, A.; Kuzmanovic, M.; Marcé, R.; Nadal, M.; Riera, J.M.; Barceló, D. Emission factor estimation of ca. 160 emerging organic microcontaminants by inverse modeling in a Mediterranean river basin (Llobregat, NE Spain). *Sci. Total Environ.* **2015**, *520*, 241–252, doi:10.1016/j.scitotenv.2015.03.055.
7. Ramirez, R.; Martí, V.; Darbra, R.M. Environmental Risk Assessment of Silver Nanoparticles in Aquatic Ecosystems Using Fuzzy Logic. *Water* **2022**, *14*, 1885, doi:10.3390/w14121885.
8. EU Annexes 1-6 to the Proposal of Directive (EU) COM(2022) 540 final Available online: https://eurlex.europa.eu/resource.html?uri=cellar:d0c11ba6-55f8-11ed-92ed%0D%0A01aa75ed71a1.0001.02/DOC_2&format=PDF (accessed on Mar 14, 2023).
